# NPSV-deep: a deep learning method for genotyping structural variants in short read genome sequencing data

**DOI:** 10.1093/bioinformatics/btae129

**Published:** 2024-03-05

**Authors:** Michael D Linderman, Jacob Wallace, Alderik van der Heyde, Eliza Wieman, Daniel Brey, Yiran Shi, Peter Hansen, Zahra Shamsi, Jeremiah Liu, Bruce D Gelb, Ali Bashir

**Affiliations:** Department of Computer Science, Middlebury College, Middlebury, VT 05753, United States; Department of Computer Science, Middlebury College, Middlebury, VT 05753, United States; Department of Computer Science, Middlebury College, Middlebury, VT 05753, United States; Department of Computer Science, Middlebury College, Middlebury, VT 05753, United States; Department of Computer Science, Middlebury College, Middlebury, VT 05753, United States; Department of Computer Science, Middlebury College, Middlebury, VT 05753, United States; Department of Computer Science, Middlebury College, Middlebury, VT 05753, United States; Google, Mountain View, CA 94043, United States; Google, Mountain View, CA 94043, United States; Mindich Child Health and Development Institute and the Departments of Pediatrics and Genetics and Genomic Sciences, Icahn School of Medicine at Mount Sinai, New York, NY 10029, United States; Google, Mountain View, CA 94043, United States

## Abstract

**Motivation:**

Structural variants (SVs) play a causal role in numerous diseases but can be difficult to detect and accurately genotype (determine zygosity) with short-read genome sequencing data (SRS). Improving SV genotyping accuracy in SRS data, particularly for the many SVs first detected with long-read sequencing, will improve our understanding of genetic variation.

**Results:**

NPSV-deep is a deep learning-based approach for genotyping previously reported insertion and deletion SVs that recasts this task as an image similarity problem. NPSV-deep predicts the SV genotype based on the similarity between pileup images generated from the actual SRS data and matching SRS simulations. We show that NPSV-deep consistently matches or improves upon the state-of-the-art for SV genotyping accuracy across different SV call sets, samples and variant types, including a 25% reduction in genotyping errors for the Genome-in-a-Bottle (GIAB) high-confidence SVs. NPSV-deep is not limited to the SVs as described; it improves deletion genotyping concordance a further 1.5 percentage points for GIAB SVs (92%) by automatically correcting imprecise/incorrectly described SVs.

**Availability and implementation:**

Python/C++ source code and pre-trained models freely available at https://github.com/mlinderm/npsv2.

## 1 Introduction

Structural variants (SVs), defined here as variants ≥ 50 bp, play a causal role in numerous diseases but can be difficult to detect and accurately genotype (determine zygosity) with short-read genome sequencing data (SRS) (Alkan *et al.* 2011, Weischenfeldt *et al.* 2013, Guan and Sung 2016). While many tools combine SV discovery and genotyping (Kosugi *et al.* 2019, Mahmoud *et al.* 2019), our focus is ‘stand-alone’ genotyping of SVs previously reported in the literature, detected by other technologies, e.g. long-read sequencing (LRS), or identified by upstream discovery tools. Stand-alone genotyping is used in workflows that genotype SVs from previous (possibly LRS) population studies in SRS samples, integrate multiple SV discovery tools, and/or combine SV call sets from multiple individuals into a coherent dataset with genotypes for all SVs across all samples (Chander *et al.* 2019).

Since SVs are similar in size to or larger than the SRS read length, SV genotypes must often be inferred indirectly from read depth, discordant read-pairs, split reads and other features in the SRS data (Alkan *et al.* 2011). Thus, there remains a substantial gap between the genotyping accuracy for small variants and SVs. LRS detects 2- to 3-fold more SVs than SRS (Audano *et al.* 2019, Ebert *et al.* 2021), showing how much more there is to learn about the true SV burden. However, LRS data are only available for a subset of samples (Goodwin *et al.* 2016). Improved SRS SV genotyping accuracy can leverage the detection capabilities of LRS and other technologies in the much larger and still growing set of SRS genomes (Audano *et al.* 2019, Chander *et al.* 2019, Ebert *et al.* 2021).

Existing SRS SV genotyping methods (Rausch *et al.* 2012, Chiang *et al.* 2015, Handsaker *et al.* 2015, Spies *et al.* 2015, Antaki *et al.* 2018, Audano *et al.* 2019, Chen *et al.* 2019, Eggertsson *et al.* 2019, Belyeu *et al.* 2021, Hickey *et al.* 2020) (see Chander *et al.* for a comparison) typically employ statistical models, rule-based and/or machine learning approaches to aggregates of SV evidence, e.g. relative read-depth or counts of ‘alternate’ reads. Machine learning-based methods are typically trained on a small number of genome-wide classes, e.g. the three possible diploid genotypes (Antaki *et al.* 2018, Belyeu *et al.* 2021). These approaches make the simplifying but often invalid assumptions that reads are independent (and, thus, can be aggregated) (Poplin *et al.* 2018) and/or that similar evidence is observed across SVs (i.e. ‘test’ and ‘training’ data are similar). The different types of SVs, range of SV sizes, different genomic contexts and different sequencers and alignment pipelines all influence the available evidence for predicting the genotype. These challenges motivate sample- and SV-specific approaches that can leverage the relationships among all reads/bases.

DeepVariant (Poplin *et al.* 2018) demonstrated state-of-the-art accuracy by recasting small variant-calling as an image analysis problem that can exploit advances in deep learning. DeepVariant replaced existing statistical models and aggregate features with a deep neural network (DNN) trained to predict the genotype from a multi-channel pileup image of overlapping reads (mimicking how human experts review SRS data). Thus, the model can learn from all the reads together. Similar image-based approaches have since been applied to SV DEL genotyping (Bai *et al.* 2021, Belyeu *et al.* 2021) and SV calling (Cai *et al.* 2019, Popic *et al.* 2023) in SRS data. DNNs can fail when making predictions for data without similar labeled examples and, thus, can require large training datasets (Marcus 2018). Compared to small variants, however, there are many-fold fewer labeled SVs available to use as training data for a larger space of variation. Limited training data, e.g. has restricted some prior DNN-based SV genotypers to DELs only (Belyeu *et al.* 2021). We propose that SV genotyping is more analogous to facial recognition, which has many (potentially unbounded) outputs with few examples per output, than to image classification, with its fixed number of outputs and many examples per output (e.g. the DeepVariant approach).

We present NPSV-deep, a novel method that formulates SV genotyping as an image similarity problem amenable to data-efficient metric learning approaches (Hadsell *et al.* 2006, Koch *et al.* 2015, Wang *et al.* 2021). NPSV-deep predicts the SV genotype based on the similarity between actual and simulated SRS data. SRS simulation has previously been used as a source of labeled training data (Chu *et al.* 2014, Linderman *et al.* 2021, Popic *et al.* 2023); here, we use simulation to inform the expected evidence during genotyping (inference). Prior work showed SRS simulation can be used to automatically create more accurate SV-specific genotypers (using fixed aggregate features) (Linderman *et al.* 2021). Instead of attempting to model the complex and interconnected effects of the SV, genomic region, sequencer, and alignment pipeline on the observed SV evidence, SRS simulation can provide a prediction of the expected evidence. We train a DNN for the simpler task of image similarity (are the two pileup images the same SV/genotype), but by using simulation to generate labeled images during genotyping, we can apply that DNN to accurately predict SV genotypes for any variant, not just those previously observed. Compared to simulation-based approaches that use fixed aggregate features (Linderman *et al.* 2021), NPSV-deep’s DNN incorporates more evidence (from all overlapping reads) while learning those features where the simulated data is most informative.

We report a rigorous evaluation of NPSV-deep across multiple truth sets for the HG002 and NA12878 samples. We compare NPSV-deep to similar stand-alone SV genotyping tools, including NPSV, our previous, non-DNN, simulation-based approach. We show that NPSV-deep achieves state-of-the-art genotype concordance for the Genome-in-a-Bottle call set (Zook *et al.* 2020), consistently achieves similar or improved genotyping accuracy across multiple call sets and samples for both DEL and INS SVs, is robust to imprecise SV descriptions, and can accurately identify putative *de novo* SVs in trios.

## 2 Materials and methods

**Figure 1.**
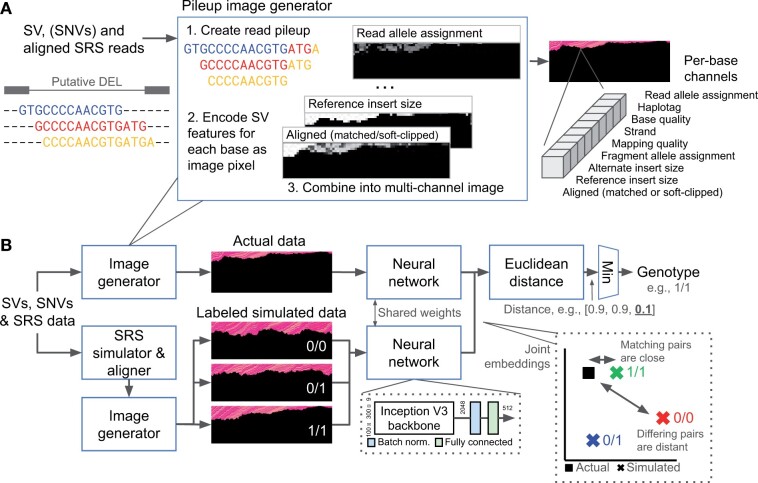
NSPV-deep image representation and dataflow. (A) NPSV-deep generates 100×300×9 image representations of the coverage pileup of SRS reads in the SV region (rendered here as composite images). Nine SV features (right) are encoded for each sequenced base as image pixels (all black pixels are the absence of a sequenced base). For larger SVs, the image is compressed to the fixed size expected by the DNN. (B) The inputs to NPSV-deep are the SRS data, putative SVs and, optionally, phased SNVs. For each SV and possible genotype, NPSV-deep generates and aligns 1+ simulated replicates using a SRS simulator and pipeline configured to match the actual sequencing data. The genotype is determined from the closest pair of embeddings of the actual and labeled simulated data. The joint embeddings are generated using identical DNNs (inset, bottom), trained with contrastive loss using 30 samples from the HGSVC2 dataset. Contrastive loss minimizes the distance in embedding space between actual and simulated pairs with matching genotypes and maximizes the distance between differing pairs (inset, right).

Figure 1 shows the NPSV-deep image representation and dataflow. The inputs are the aligned reads (BAM/CRAM), termed the ‘actual’ data, sequence-resolved deletion (DEL) and insertion (INS) SVs (VCF), and optional phased SNVs (VCF). Other SV types are not currently supported. The output is a copy of the input SVs with predicted genotypes.

### 2.1 Image generation

NPSV-deep generates multi-channel coverage pileup image representations of the SRS data as 100 × 300 × 9 tensors (Fig. 1A). The images encode nine SV features for each sequenced base, including (re-)alignment, insert size, quality and haplotype information, as pixels starting the ‘top’ of the image (all black pixels are the absence of a sequenced base). The Supplemental Methods describe the channels in more detail. NPSV-deep generates fixed-height pileup images spanning the entire event. Due to the large number of reads spanning larger SVs, the pileup images are generated as coverage histograms. Each pixel is initially a single sequenced base; the resulting variable-width image is compressed to the fixed size expected by the DNN.

### 2.2 Neural network architecture and training

As described previously (Linderman *et al.* 2021), for each SV and possible genotype, NPSV-deep generates 1+ synthetic SRS datasets using an SRS simulator (Huang *et al.* 2012) and alignment pipeline configured to match the actual data. NPSV-deep implements a paired DNN (Koch *et al.* 2015) to genotype SVs based on the similarity of the image representations generated from the actual and simulated data. Pairs of actual and simulated images are transformed by identical DNNs into a ‘joint’ high-dimensional embedding space (d=512) in which the actual data and the simulated data with the matching genotype are similar, i.e. the distance between their embeddings approaches 0, and dissimilar otherwise (Fig. 1B, right inset). We determine the final genotype from the closest pair among the three possible genotypes: homozygous reference, heterozygous and homozygous alternate (0/0, 0/1, 1/1 in a bi-allelic VCF entry). For multi-allelic SVs, the genotype is determined from the most similar pair among all combinations of alleles and genotypes.

We trained the NPSV-deep using contrastive loss (Hadsell *et al.* 2006). Contrastive loss (Equation 1, sketched Fig. 1B inset):

(1) $$\begin{array}{c} L(Y,D)=Y\cdot D^{2}+(1-Y)\cdot \max{\left(m-D,0\right)}^{2} \end{array}$$

minimizes the distance (*D*) in the embedding space between actual and simulated pairs with matching genotypes (Y=1) and maximizes (up to a threshold, *m*) the distance between differing pairs. The training data consisted of the HGSVC2 freeze 3 SV call set (Ebert *et al.* 2021), initially 106 768 SVs (INS/DEL, 50 bp to 15 Mbp in size) identified in haplotype-resolved assemblies of 32 unrelated diverse individuals, and high-coverage SRS of the 1000 Genomes (1KG) cohort (Byrska-Bishop *et al.* 2022). We removed the evaluation samples, HG002/NA24385 and NA12878, and 1171 SVs (1.1%) private to those samples (where the only alternate alleles were reported in HG002 or NA12878). The derived training call sets contained 30 samples with 28 555–30 794 DEL and 40 269–43 003 INS SVs per sample. For each SV, we generated 3+ image pairs comprising a common image of the actual data and images of 1+ simulated replicates of each genotype, labeled 1 if the simulated genotype matches the HGSVC2-reported genotype and 0 otherwise. We combine multiple models into an ensemble by averaging the distances independently predicted for each image pair.

Figure 1 shows composite images for a 822-bp homozygous alternate DEL. This SV is a deletion of a single copy of a repeat (shown in Supplementary Fig. S1). As a result of that repetitive context, no alternate spanning pairs or alternate allele reads were identified. Methods that use the actual data alone can incorrectly predict a homozygous reference genotype. However, as shown by the visual similarity of the actual and simulated images (including the individual channels rendered in Supplementary Fig. S1), the observed evidence is most consistent with a homozygous alternate genotype and not homozygous reference as might be expected. NPSV-deep reports homozygous alternate as the most similar actual-simulated pair, correctly genotyping this SV.

### 2.3 Evaluation

We evaluated the genotypers using the Genome in a Bottle (GIAB) v0.6 SV callset in HG002/NA24385 (GRCh37) and the Polaris 2.1 (https://github.com/Illumina/Polaris) and HGSVC2 freeze 3 SV callsets in NA12878 (GRCh38). SVs < 50 bp, > 15 Mbp, filtered (except for ‘LongReadHomRef’ SVs in GIAB), or with missing genotypes were excluded. Unless otherwise noted, analyses used the truth sets as the input SVs. Supplementary Table S2 shows the breakdown of genotypes in each dataset.

We genotyped the HG002 SVs in 2×148 PCR-free SRS data (mean coverage 263× for HG002). Unless otherwise noted, analyses used a fixed subset of the data (25.5×, 24.7×, and 20.4× for HG002, HG003, and HG004, respectively). We aligned the SRS data to GRCh37 and performed SNV calling and SV discovery (with Lumpy and Manta) using the BCBio pipeline (v1.2.8) with the default BWA/GATK configuration (Layer *et al.* 2014, Chen *et al.* 2016, Chapman *et al.* 2021). We genotyped the NA12878 SVs in the Platinum Genomes 2×100 SRS data (mean coverage 50.5×) aligned to GRCh38 with the same BCBio pipeline (Eberle *et al.* 2017). We performed read-backed phasing of the SNVs using WhatsHap (Martin *et al.* 2016) in the SRS data.

We compared NPSV-deep to representative stand-alone SV genotyping tools using parametric algorithms: the Delly2 (v0.8.3) genotyping module (Rausch *et al.* 2012), SVTyper (v0.7.1) (Chiang *et al.* 2015), GenomeSTRiP (v2.00.1958) (Handsaker *et al.* 2015), svviz2 (2.0a3) (Spies *et al.* 2015), graph-based representations of the SV: Paragraph (v2.4a) (Chen *et al.* 2019), GraphTyper2 (v2.5.1) (Eggertsson *et al.* 2019), machine-learning based approaches with fixed features: SV2 (v1.5) (Antaki *et al.* 2018), NPSV (v1.0) (Linderman *et al.* 2021), and deep learning approaches: Samplot-ML (v0.2) (Belyeu *et al.* 2021). We executed all tools with the same input VCFs and SRS data using the default parameters. Genotype concordance is calculated with Truvari (Linderman *et al.* 2021, English *et al.* 2022) as the fraction of exactly matching genotypes among all SVs in the truth set. Nonreference concordance treats heterozygous and homozygous alternate genotypes as equivalent. To identify offset SVs, we matched GIAB Tier 1 DEL SVs to SVs called by PBSV 2.21 in PacBio CCS reads using Truvari (Chen *et al.* 2019, Linderman *et al.* 2021). See the Supplemental Methods for additional details.

## 3 Results

### 3.1 Genotyping accuracy for HG002 and NA12878

**Figure 2.**
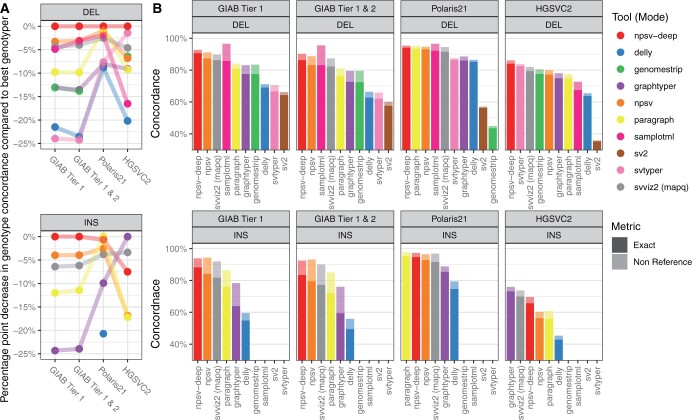
Genotyping accuracy for HG002 (GIAB) and NA12878 (Polaris 2.1, HGSVC2) SVs. (A) Summary of genotype concordance compared to the most accurate genotyper for each call set. (B) Genotype (exact) and nonreference (presence versus absence) concordance for GIAB v0.6 SVs in decreasing order of exact genotype concordance, including homozygous reference SVs, in tier 1 regions (GIAB Tier 1) and tier 1 regions and tier 2 SVs combined (GIAB Tier 1 and 2); and concordance for Polaris 2.1 and HGSVC2 call sets in NA12878.

**Figure 3.**
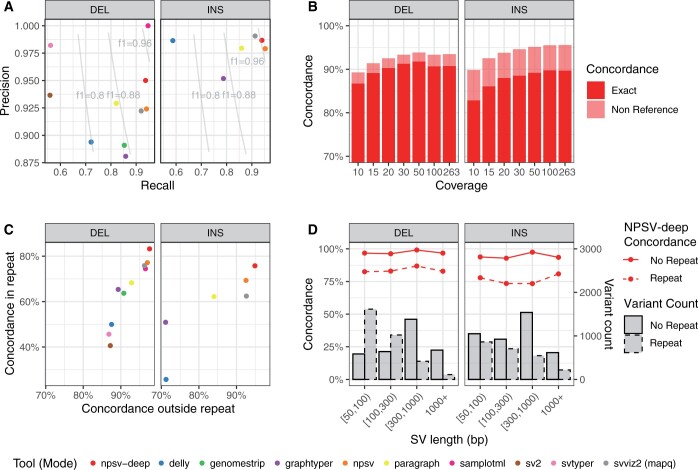
Genotyping metrics for GIAB Tier 1 SVs. (A) Event-level precision and recall (presence versus absence of SV). (B) Genotype concordance (exact) and nonreference concordance at different mean coverage levels for NPSV-deep. (C) Genotype concordance for SVs overlapping (inside) and not overlapping (outside) a TR > 100 bp (as reported in the GIAB call set). (D) NPSV-deep genotype concordance for SVs stratified by size and overlap of a tandem repeat (TR) > 100 bp. The bars show SV counts.

Figure 2 shows genotyping accuracy for the GIAB, Polaris 2.1 and HGSVC2 SVs (values in Supplementary Tables S3 and S4). Due to randomness in the SRS simulation, NPSV-deep is nondeterministic. However, the standard deviation in genotype concordance for GIAB Tier 1 SVs across 10 runs was < 0.11 percentage points; given that small variance, we report all results for a single run. Supplementary Table S5 shows genotyping accuracy for the subset of private SVs in HGSVC2 excluded from the training data. We observe similar or improved genotyping accuracy across the different call sets, samples and variant types compared to existing SV genotypers. For the high-confidence GIAB Tier 1 SVs, NPSV-deep improves genotype concordance (nonreference) by 3.3 (−3.8) and 4.0 (−0.5) percentage points for DEL and INS SVs, respectively, compared to the best tool for each metric. Figure 3A shows event-level (presence versus absence of an SV) precision and recall for the GIAB Tier 1 SVs; Supplementary Fig. S2 shows the same metrics for the other call sets.

The evaluation call sets have different distributions of genotypes (Supplementary Table S2). The Polaris 2.1 and HGSVC2 truth sets were derived from multi-sample call sets and so are enriched for homozygous reference genotypes (SVs present in other samples but not in NA12878). Thus, tools with higher precision but lower recall will achieve high accuracy for those datasets but not for the more balanced GIAB call set. As a result of heterogeneity in the population (Liao *et al.* 2023) [and/or imprecision in SV discovery (Kirsche *et al.* 2023)], the multi-sample HGSVC2 call set has numerous similar or overlapping SVs, only a fraction of which are present in any specific sample. Reference SVs similar to or overlapping a nonreference SV are often ‘overcalled,’ increasing false positive rates and reducing genotyping accuracy. Techniques for increasing accuracy for overlapping SVs are described further below.

Figure 3B shows the genotyping accuracy for the GIAB Tier 1 SVs for different mean coverage levels (downsampled from 263× coverage). Accuracy generally increases with increasing coverage until 100×, the height of the image and thus the threshold at which the image generator randomly downsamples the SRS data to fit within image window. The default height was chosen to provide sufficient range for typical GS datasets with coverage of 30–40×, while reducing computational requirements.

Figure 3C shows genotyping accuracy for the GIAB Tier 1 SVs overlapping and not overlapping repetitive regions (tandem repeats > 100 bp). As expected, we observe reduced accuracy for SVs overlapping repetitive regions. Figure 3D shows genotyping accuracy for NPSV-deep for (non) repetitive SVs further stratified by SV length (SVLEN). Accuracy is generally consistent across different size categories. Supplementary Fig. S3 expands Fig. 3D with select comparison genotypers and Tier 1 and 2 SVs.

Supplementary Fig. S4 shows the genotyping accuracy for the GIAB SVs when SNVs are not provided, and for different sources of SNVs. We observed a small overall improvement in genotype concordance for GIAB SVs (0.07–0.15 percentage points) when incorporating phased SNVs, driven by improved accuracy for DEL SVs.

To investigate using NPSV-deep to genotype SVs discovered in SRS data, we re-genotyped SVs identified with Lumpy/SVTyper and Manta in HG002. Table 1 shows genotype concordance using the discovery call set instead of the truth set as the SV input. To focus on genotyping accuracy, we exclude SVs that were not detected by the SV discovery tools. NPSV-deep achieves improved accuracy compared to the genotypes reported by the upstream caller.

**Table 1.** NPSV-deep genotyping accuracy for GIAB Tier 1 regions using discovery SVs as the input.^a^

Caller	Type	Discovery recall (%)	Caller genotyping (%)		NPSV-deep genotyping (%)	
			Concordance	Nonreference concordance	Concordance	Nonreference concordance
Lumpy	DEL	28.8	87.5	91.2	92.4	92.7
Manta	DEL	71.5	91.0	92.9	94.2	94.9
Manta	INS	26.0	86.7	93.6	90.6	93.9

a Concordance is calculated for the subset of SVs successfully called by the discovery tool. SVs without matching variants in the truth set genotyped as homozygous reference are considered concordant.

### 3.2 Trio analysis

NPSV-deep achieves a Mendelian error rate (MER) of 4.1% (264/6416) for GIAB Tier 1 DEL and 4.1% (257/6268) INS SVs. The MER is similar to or greater than reported for the comparison genotypers (1.7%–8.8%) (Linderman *et al.* 2021). The two DEL Mendelian errors (ME) explicitly reported in the GIAB Tier 1 SVs (as a ‘likely *de novo* deletion’ and a deletion ME in a locus known to undergo somatic rearrangement) (Zook *et al.* 2020) were also reported as *de novo* by NPSV-deep. NPSV-deep reported these SVs to be the two most confident DEL MEs, as determined by the minimum genotype probability (softmax of distances) across all trio members, indicating that MEs could be specifically filtered.

### 3.3 Refining offset SVs

**Figure 4.**
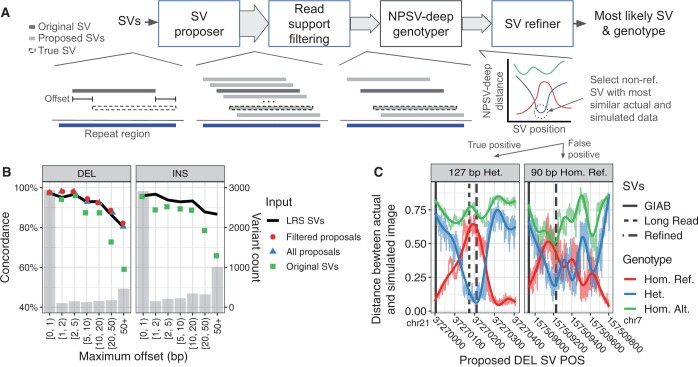
Genotyping offset SVs. (A) Example of offset SV and SV refining workflow. The proposer generates alternate SV descriptions overlapping repetitive regions, which are then filtered for read support and genotyped with NPSV-deep. Distant proposals will be confidently homozygous reference; the refiner selects the nonreference minima, the SV where the nonreference simulation and actual data are most similar. SV refining is currently implemented for DELs. (B) Genotype concordance for GIAB Tier 1 nonreference SVs grouped by maximum offset between the GIAB and LRS-derived breakpoints for NPSV-deep genotyping the LRS-derived SV descriptions (line), the SVs as originally described in the GIAB VCF (green squares), and with SV refining (blue triangles, red circles). The bars show the SV counts. (C) Example correctly refined 127-bp heterozygous DEL SV (left) and an incorrectly refined 90-bp homozygous reference DEL SV (right). The colored lines show the distances for all possible starting SV positions within the repetitive region (all proposals), with the vertical lines showing the original GIAB SV position (solid), LRS derived SV position (dotted), and the position selected by the refiner (dashed).

Manual review of incorrectly genotyped SVs indicated that offset SV descriptions, differences between the putative and true SV breakpoints/alleles (Fig. 4A, left), were a contributing factor to genotyping errors in repetitive regions and more generally (Chen *et al.* 2019, Linderman *et al.* 2021). Imprecise or incorrect SV descriptions result in inconsistent evidence and reduced genotyping accuracy.

Figure 4B shows the impact of offset breakpoints. We matched the GIAB Tier 1 nonreference SVs to the corresponding SVs called by PBSV (https://github.com/PacificBiosciences/pbsv) in LRS data (4124/4203 DEL and 5174/5443 INS SVs matched). Making the assumption that the PBSV breakpoints are correct, we infer the maximum offset from the distance between the GIAB and PBSV breakpoints. Accuracy remains above 86% for DEL and INS SVs offset by up to 20 bp, but as expected, we observe a decrease in genotyping accuracy at larger offsets (Fig. 4A green squares). Using the LRS-derived SVs instead of the GIAB SVs as the input to NPSV-deep mitigates that decrease (Fig. 4B, solid line), indicating that correcting the SV description can improve genotyping accuracy.

To increase genotyping accuracy for SVs with offset breakpoints, the previous NPSV genotyper incorporated ‘SV refining’ to automatically correct the SV description using the SRS data alone (Linderman *et al.* 2021). Motivated by the hypothesis that the actual and simulated data are most similar for the true SV description and genotype, NPSV selected the SV with the closest actual and simulated SV evidence among proposed alternate SV descriptions. NPSV showed modest improvements (< 1 percentage point) in genotyping accuracy with SV refining but used a fixed distance metric applied to only 10 proposals per SV (due to computational limitations). We hypothesized that NPSV-deep’s learned similarity metric would be more effective, while its reduced computational requirements would enable a more comprehensive set of proposals.

We implemented the ‘SV refining’ workflow shown in Fig. 4A. We propose and genotype alternate descriptions for SVs overlapping repetitive regions, up to all possible starting positions within the repetitive region. The SV refiner selects the most likely SV representation based on the minimum distance between the actual and simulated data predicted by NPSV-deep across all proposed SVs.

Figure 4C shows the distances for proposed SVs at each potential SV starting position for an example successfully refined (‘true positive’) 127-bp heterozygous DEL SV (left) and an incorrectly refined (‘false positive’) 90-bp homozygous reference DEL SV (right). The GIAB reported position is at the left (solid vertical line). For the correctly refined SV, the LRS-derived position is 169 bp downstream (dotted vertical line). The SV refiner selected the SV and genotype at the distance minima indicated with dashed vertical line, correctly reporting a heterozygous genotype and a location that is more similar to the LRS position for this SV. Since we expect distant proposals to be confidently homozygous reference, the refiner selects the nonreference minima. To suppress noise in the distance estimate, an exponentially weighted moving average is applied to each genotype. In the incorrectly genotyped SV (Fig. 4C, right), the refiner creates a false-positive call by selecting the heterozygous minima, even though no SV is present in this region. Increasing the specificity of the refining process is a focus of ongoing work.

Figure 4B shows the genotype concordance after applying SV refining. SV refining increases overall genotype (nonreference) concordance by 1.5 (1.7) percentage points for Tier 1 DEL SVs by reducing false negatives (failure to identify nonreference genotypes). The refined SVs match the genotyping concordance when using the LRS-derived SVs as the input call set. Testing all possible SV starting positions for the 8435 GIAB DEL SVs required genotyping > 7 million SVs (blue triangles). Incorporating *k*-mer-based filtering to discard proposals without supporting SRS data reduced the input to 2.13 million SVs while modestly improving accuracy by reducing false positive selections (red circles).

Population-scale datasets often have groups of similar or overlapping SVs. If we assume that any individual only has one nonreference SV in a local genomic region, the problem of selecting the true nonreference SVs in a population call set is analogous to selecting among proposed alternative SVs in the refining workflow. Applying the refining step improves genotyping accuracy for the HGSVC2 call set compared to NSPV-deep alone, achieving the highest concordance among all the comparison genotypers for both DEL and INS SVs (Supplementary Fig. S5).

### 3.4 Computational requirements

NPSV-deep requires 146 min and 34.7 GB of memory to genotype the *n*=16 866 GIAB SVs on a 36-core compute node. The preprocessing step to compute sequencing statistics required 2.3 min (25.5× BAM file). Supplementary Table S6 lists the execution time and memory for all tools running on the same system. At present, genotyping is CPU-only. NPSV-deep is distributed with the pre-trained models used in this evaluation. Training new models required 10–100s of hours using a NVIDIA V100 GPU depending on the choice of hyperparameters.

## 4 Discussion

NPSV-deep is a novel stand-alone SV genotyper that recasts SV genotyping as an image similarity problem that can leverage ongoing advances in machine learning with DNNs. Instead of predicting the SV genotype from the actual SRS data alone (‘direct classification’), NPSV-deep predicts the SV genotype from the most similar pair of pileup images generated from the actual and simulated SRS data.

NPSV-deep consistently achieves similar or better genotyping accuracy than comparison genotypers for both DEL and INS SVs across multiple call sets and samples (Fig. 2). For the GIAB Tier 1 SVs, NPSV-deep improves genotype concordance (nonreference) by 3.3 (−3.8) and 4.0 (−0.5) percentage points for DEL and INS SVs respectively, representing a 25% reduction in genotyping errors. The evaluation incorporated similar stand-alone SV genotypers [versus SV discovery tools with integral genotyping (Cai *et al.* 2019, Popic *et al.* 2023)], with pre-trained models available (Bai *et al.* 2021), capable of genotyping a wide range of SVs, e.g. not just those sourced from phased assemblies (Ebler *et al.* 2022), in a single sample. NPSV-deep demonstrated improved genotyping accuracy starting with both the truth set SVs and the output of SV discovery tools (Table 1). NPSV-deep correctly and specifically identified the putative *de novo* DEL SVs reported by GIAB.

Compared to direct classification approaches, NPSV-deep incorporates more information, i.e. labeled simulations of that specific SV, into the genotyping process. SRS simulation is necessarily imperfect (Cameron *et al.* 2019). However, the metric of interest is genotyping accuracy, not simulation fidelity. Since NPSV-deep’s paired DNN learns features shared by the actual and simulated data, NPSV-deep is less sensitive to simulation fidelity compared to methods that use fixed, aggregate features (e.g. the previous NPSV genotyper).

NPSV-deep is robust to small offsets in the putative SV description, maintaining a genotype concordance of 87.3% and 88.7% for offsets up to 20 bp (Fig. 4). As noted previously, making SV features more robust to incorrect SV descriptions could improve genotyping accuracy, but genotyping an incorrectly described SV as nonreference would not be strictly accurate as that specific alternate allele is absent (Linderman *et al.* 2021). We implemented a form of ‘SV refining’ that attempts to select a more accurate description of the SV during genotyping based on the similarity between the actual and simulated data across proposed alternate SV descriptions. In NPSV-deep, SV refining further improved genotype (nonreference) concordance for GIAB Tier 1 SVs by 1.5 (1.7) percentage points, eliminating the accuracy gap between using the GIAB SVs as the input to the genotyper and LRS-derived SVs (Fig. 4B).

NSPV-deep is limited to sequence-resolved variants so that it can simulate the putative SV alleles. It does not support ‘position independent’ SVs, e.g. duplications with arbitrary copy numbers. NPSV-deep currently supports DEL and INS variants (for which there are available training data), although the underlying approach could be extended to any sequence resolved SV (e.g. inversions). In default usage (without refining), NPSV-deep currently treats each VCF record independently and does not attempt to automatically combine records (e.g. to create compound heterozygous SVs or multiple SVs on the same haplotype).

We hypothesize that NPSV-deep’s simulation-based approach, which generates an empirical estimate of the observed data, will be particularly useful for those complex SVs, which are difficult to genotype (Cameron *et al.* 2019, Zook *et al.* 2020), and for which there are even fewer similar high-quality training examples available. Improving NPSV-deep’s similarity metric for complex SVs and extending SV refining to synthesize and select among complex SVs is a focus of ongoing work. More accurate SV refining could increase genotyping accuracy for offset/imprecise/incompletely described SVs, reduce false positive nonreference genotypes among similar or overlapping SVs in population-scale call sets (Supplementary Fig. S5) and ultimately improve SV discovery by more precisely selecting among candidate SVs.

## Supplementary Material

btae129_Supplementary_Data

## Data Availability

The HGSCV2 call set is available at https://www.internationalgenome.org/data-portal/data-collection/hgsvc2. The 1KG SRS training data is available at https://www.internationalgenome.org/data-portal/data-collection/30x-grch38. The Syndip callset is available at https://github.com/lh3/CHM-eval. The GIAB call set is available at https://ftp-trace.ncbi.nlm.nih.gov/giab/ftp/release/AshkenazimTrio/HG002_NA24385_son/NIST_SV_v0.6 and the SRS data at https://ftp-trace.ncbi.nlm.nih.gov/ReferenceSamples/giab/data//AshkenazimTrio/HG002_NA24385_son/NIST_HiSeq_HG002_Homogeneity-10953946/HG002_HiSeq300x_fastq, https://ftp-trace.ncbi.nlm.nih.gov/ReferenceSamples/giab/data//AshkenazimTrio/HG003_NA24149_father/NIST_HiSeq_HG003_Homogeneity-12389378/HG003_HiSeq300x_fastq, and https://ftp-trace.ncbi.nlm.nih.gov/ReferenceSamples/giab/data//AshkenazimTrio/HG004_NA24143_mother/NIST_HiSeq_HG004_Homogeneity-14572558/HG004_HiSeq300x_fastq. The Polaris 2.1 call set is available at https://github.com/Illumina/Polaris. The NA12878 SRS data are available in the European Nucleotide Archive at https://ftp.sra.ebi.ac.uk/vol1/fastq/ERR194/ERR194147 via project PRJEB3381.
