# Supplementary material for: NPSV-deep: a deep learning method for genotyping structural variants in short read genome sequencing data
Source: Bioinformatics. 2024 Mar 5;40(3):btae129. doi: 10.1093/bioinformatics/btae129 (PMC10955255; doi:10.1093/bioinformatics/btae129)
Supplement: btae129_Supplementary_Data [file btae129_supplementary_data.pdf]

NPSV-deep: A deep learning method for genotyping structural variants in genome sequencing data

Supplemental Methods and Results

Michael D. Linderman[1]*, Jacob Wallace[1], Alderik van der Heyde[1], Eliza Wieman[1], Daniel Brey[1], Yiran Shi[1], Peter Hansen[1], Zahra Shamsi[2], Jeremiah Liu[2], Bruce D. Gelb[3], Ali Bashir[2]

[1]Department of Computer Science, Middlebury College, Middlebury, VT, USA
[2]Google, Mountain View, CA, USA
[3]Mindich Child Health and Development Institute and the Departments of Pediatrics and Genetics and Genomic Sciences, Icahn School of Medicine at Mount Sinai, New York, NY, USA

*Corresponding Author:

Michael D. Linderman
mlinderman@middlebury.edu
Department of Computer Science
Middlebury College
14 Old Chapel Road
Middlebury, VT 05753

Supplemental Methods **3**
NPSV-deep genotyping algorithm 3
Image generation 3
Neural network architecture and training 4
SV refining 5
Evaluation 5
Supplemental Results **8**
References **18**

Supplemental Methods

NPSV-deep genotyping algorithm

NPSV-deep is a Python-based software tool that performs image generation, model training, and genotyping for sequence resolved SV insertions (INS) and deletions (DEL). The inputs are the aligned reads (BAM/CRAM file), a VCF file of putative SVs, and a VCF file of phased SNVs for the same sample. NPSV-deep produces a copy of the input SV VCF with predicted SV genotypes and associated metadata.

Image generation

The NPSV-deep image representations (Figure 1A) are 9-channel tensors incorporating the alignment, allele, insert size, quality and haplotype information described below and Supplemental Table 1. The image region is centered on and spans the entire event plus a flanking region. During image generation, each pixel is a single sequenced base. Reads are rendered in sorted order of genomic coordinates. Due to the large number of reads spanning larger SVs, the pileup images are generated as coverage histograms, i.e., horizontally adjacent pixels may be derived from different reads. If the coverage would exceed the maximum image height, reads are randomly downsampled so that the total coverage is less than the number of image pixels. The resulting variable-width image (SV width plus flanks) is compressed to a fixed size (default of 100×300).

Base alignment, e.g. match, mismatch, soft clip, is derived from the CIGAR string of the original alignments. Reads are assigned to the reference or alternate allele by realigning the read to the reference and alternate sequence (derived from the putative SV) using BWA[1] (via SeqLib[2]) and a scoring metric adapted from svviz2[3]. Only reads originally aligned within some flanking distance of the image region are realigned. We haplotag reads with WhatsHap[4] and the phased input SNVs (provided as an optional input) to identify SV features observed only on a single haplotype (indicative of a heterozygous genotype)[5,6]. We compute reference and alternate insert size z-scores from the SV length and an estimate of the insert size distribution. Since the fragment insert could span the entire image region and, thus, not be represented by any bases in the image, we include “insert” pixels that encode the insert size for spanning fragments with reads outside the image region. Enumerated values, e.g., strand, are mapped to fixed pixel values, while continuous values, such as quality scores are linearly mapped to pixel ranges. Pixels with all zeros (black in the composite images in Figure 1) are the absence of a sequenced base.

The same image generation procedure is applied to the actual and simulated SRS data. Similar to NPSV[7], the simulated data is generated with the ART SRS simulator[8] configured to match the original sequencing data (sequencer error model, read length, coverage, insert size distribution) and aligned with the same pipeline as the actual data. In this evaluation we align reads with BWA-MEM[1] and mark duplicates with samblaster[9] to mimic the BCBio pipeline applied to the actual data[10]. To enable haplotagging, we insert phased SNVs into the simulated haplotypes (no

relationship between the SV and SNV alleles is assumed). A preprocessing step computes the mean, per-chromosome coverage and insert size distribution with bedtools[11], SAMtools[12] and indexcov[13] to inform the SRS simulation and image generation.

Neural network architecture and training

Figure 1B shows the genotyping model. NPSV-deep implements a paired network to transform the paired images (100×300×9) of actual and simulated data using identical DNNs into a joint embedding space (d = 512)[14]. The genotype label is determined from the label of the simulated data most similar to the actual data (smallest Euclidean distance). For multi-allelic SVs, the genotype is determined from the smallest distance across all possible combinations of alleles and genotypes. The neural network, training and inference were implemented with Tensorflow v2.8[15].

The DNN backbone is shown in the Figure1A inset. The backbone is the Inception V3 image classification network with the original last layer removed, connected to a single fully connected projection layer[16]. We implement a standard training loop using the Adam optimizer and a fixed learning rate (0.004). The gradients for the paired backbone networks are additive due to the shared weights[14]. Due to the structural differences between the pileup images and the ImageNet dataset used to originally train the InceptionV3 model, we do not implement transfer learning and instead train the entire DNN (including Inception V3) from random initial weights.

We train the genotyping model using a 30 sample subset of the HGSVC2 sequence-resolved phased SV and SNV call set with the NA12878 and HG002/NA24385 samples and their private SVs excluded[17]. The SRS data is sourced from the high-coverage 1000 Genomes dataset[18]. The multi-sample HGSVC2 call set contains multiple similar and overlapping SVs in some regions. To reduce instances of putative homozygous reference SVs partially overlapping non-reference SVs we filtered the training call set to isolate non-reference SVs. For each sample, we grouped overlapping SVs. Groups with only reference SVs were retained as is, groups with a single non-reference SV were collapsed to that non-reference SV, groups with multiple non-reference SVs were discarded. The resulting training call sets contained 28,555-30,794 DEL and 40,269-43,003 INS SVs per sample.

We trained a cohort of models for each SV type with a range of hyperparameters: 1, 3 and 5 simulated replicates, 10 and 20 epochs and with/without random simulated depth augmentation (randomly set simulated depth between 50-100% of actual depth). All models are trained to fixed endpoints without early stopping or other validation criteria. The final ensemble of five models for each variant type (DEL, INS) was selected to achieve the best combined genotyping accuracy for the Syndip validation dataset[19]. To validate model precision, we augmented the Syndip dataset with putative false positive SVs called by Lumpy[20] and Manta[21] in the Syndip SRS data.

SV refining

Figure 4A shows the SV refining workflow. For each DEL SV overlapping a repetitive region (the UCSC simple repeats track in this evaluation), the SV proposer generates alternative SV descriptions at all possible starting positions in the union of that region and any "widened" region provided in the VCF[22]. The read support filter optionally excludes any of the resulting proposed SVs that induce unique *k*-mers (k = 31) not present in SRS data. We genotype all SVs (original and proposed) using NPSV-deep. To minimize the noise observed in Figure 4C, we smooth the distances for each set of proposed SVs (original SV and size) with an exponentially weighted moving average. The SV refiner groups all proposed SVs within some distance (default of 1000 bp). For each group, we rank the proposed SVs by the minimum non-reference distance (smallest distance between actual and simulated data for heterozygous and homozygous alternate genotypes). If the smallest non-reference distance is less than that of its corresponding original SV, i.e., the actual simulated data are more similar for the proposed alternative SV than the original SV, the proposal is chosen as the "best alternate" for the group. For each original SV in the group, we update its genotype if one of its proposals was selected as the best alternate. If another SV's proposal was selected as the best alternate, we set the genotype to homozygous reference under the assumption there is only one non-reference SV per region. All SVs without a best alternate remain unchanged.

Evaluation

As noted in the main text, we evaluated the proposed and comparison genotypers using the Genome in a Bottle (GIAB) v0.6 SV callset in HG002/NA24385 (GRCh37) and the Polaris 2.1 and HGSVC2 freeze 3 SV callsets in NA12878 (GRCh38)[17,23,24] following a previously reported approach[7]. SVs less than 50 bp, greater than 15 Mbp, filtered (in the upstream dataset) except for "LongReadHomRef" SVs in GIAB (described as "Long reads supported homozygous reference for all individuals"), or with missing genotypes are excluded. GIAB SVs outside Tier 1 and Tier 2 regions were also excluded. Supplemental Table 2 shows the breakdown of genotypes in each dataset.

We executed all tools with the same input VCFs and SRS data using the default parameters. VCF FILTER annotations reduce concordance (since such SVs are treated as no-calls) and so are ignored. The default parameters may not represent the best possible configuration for each tool. Nor does this evaluation utilize all the capabilities of the comparison tools, which may support variant types that NPSV-deep does not, provide additional visualization features, or be optimized for population-scale genotyping. See the previously reported evaluation for additional descriptions of any tool-specific configuration[7].

We calculated genotyping accuracy using Truvari, modified as described previously[7,25]. Genotype concordance is calculated as the fraction of matching homozygous reference, heterozygous and homozygous alternative genotypes among all genotyped SVs in the truth set. Non-reference concordance treats heterozygous and homozygous alternate genotypes as equivalent. "No calls", i.e., "./." genotypes, in the test set are treated as incorrectly genotyped. In the discovery context (using the output of SV discovery tools as the input SVs for genotyping),

false positive SVs, i.e., variants not present in the truth set, but genotyped as homozygous reference in the test set are treated as correctly genotyped, since they are correctly indicated as not present in the individual. In the genotyping context Truvari is configured to match SVs within 20 bp, of any allele similarity, with 60% overlap, while in the discovery context (using the output of SV discovery tools as the input SVs for genotyping) Truvari is configured to match SVs within 2000 bp, of any allele similarity, with 70% overlap[23]. Mendelian errors were identified in autosomal regions using BCFTools[26]. To identify offset SVs, we matched GIAB Tier 1 DEL SVs to SVs called by PBSV 2.21[27] in PacBio CCS reads (obtained from the GIAB FTP repository) using Truvari configured to match SVs within 2000 bp, with 70% allele and size similarity and computed the maximum distance between the GIAB and PBSV breakpoints.

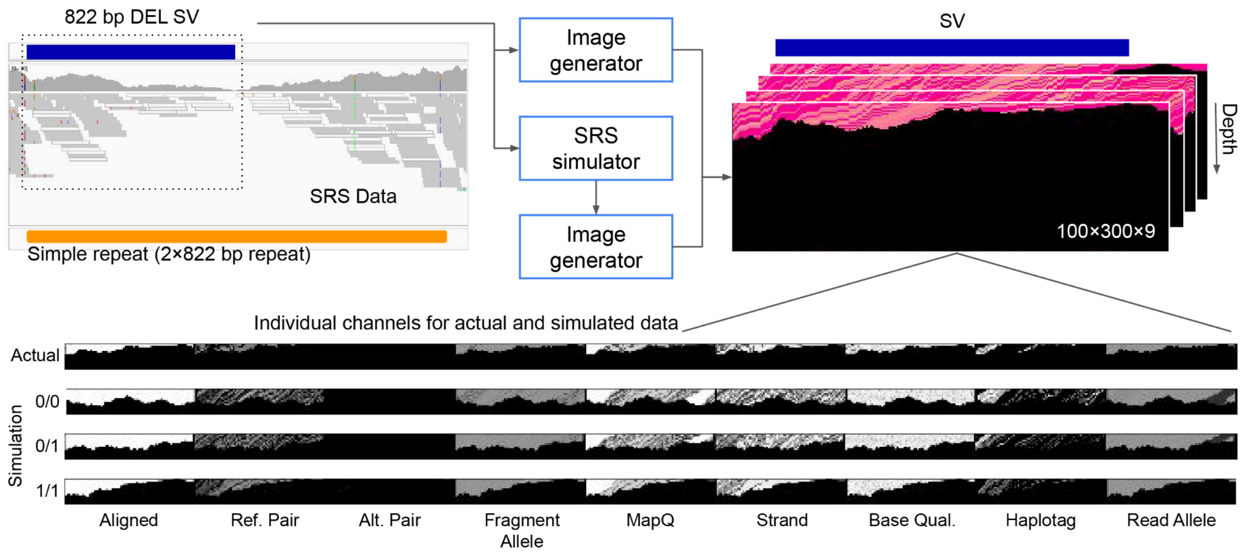

Supplemental Figure 1: Image generation for 822 bp homozygous alternate (1/1) DEL in HG002. The original SRS data is shown in an IGV[28] screenshot in the upper left. This SV is a deletion of a single copy of a repeat at 12:22129565-22130387 (GRCh37). Composite pileup coverage images generated by NPSV-deep for the actual and simulated SRS data are shown top right. The individual image channels are shown at the bottom. As a result of the repetitive context for this SV, no alternate spanning pairs or alternate allele reads were identified. However, as observed in the individual channels that evidence is consistent with a homozygous alternate genotype, and not homozygous reference as might otherwise be expected.

Supplemental Table 1: NPSV-deep image channels. Enumerated values, e.g., strand, are mapped to fixed values, while continuous values, such as quality scores, are linearly mapped to ranges.

Channel	Description
Aligned	Base is match, mismatch, soft-clipped, or insert. “Insert” bases encode the insert size for spanning fragments with reads outside the image region that would otherwise not be represented.
Reference insert size	Insert size Z-score for spanning fragments assuming a reference allele or alternate allele and an estimate of the insert size distribution.
Alternate insert size	
Fragment allele assignment	Score of read and fragment realigned to alternate allele(s) with BWA[1] (via SeqLib[2]). Fragment realignment incorporates read realignment and insert size. Realignment method adapted from svviz2[3].
Read allele assignment	
Mapping Quality	Mapping quality reported by aligner
Strand	Read strand
Base Quality	Base quality reported by sequencer
Haplotag	Read haplotags (label of 1 or 2 indicating distinct haplotypes) determined by WhatsHap[4] from phased SNVs.

Supplemental Results

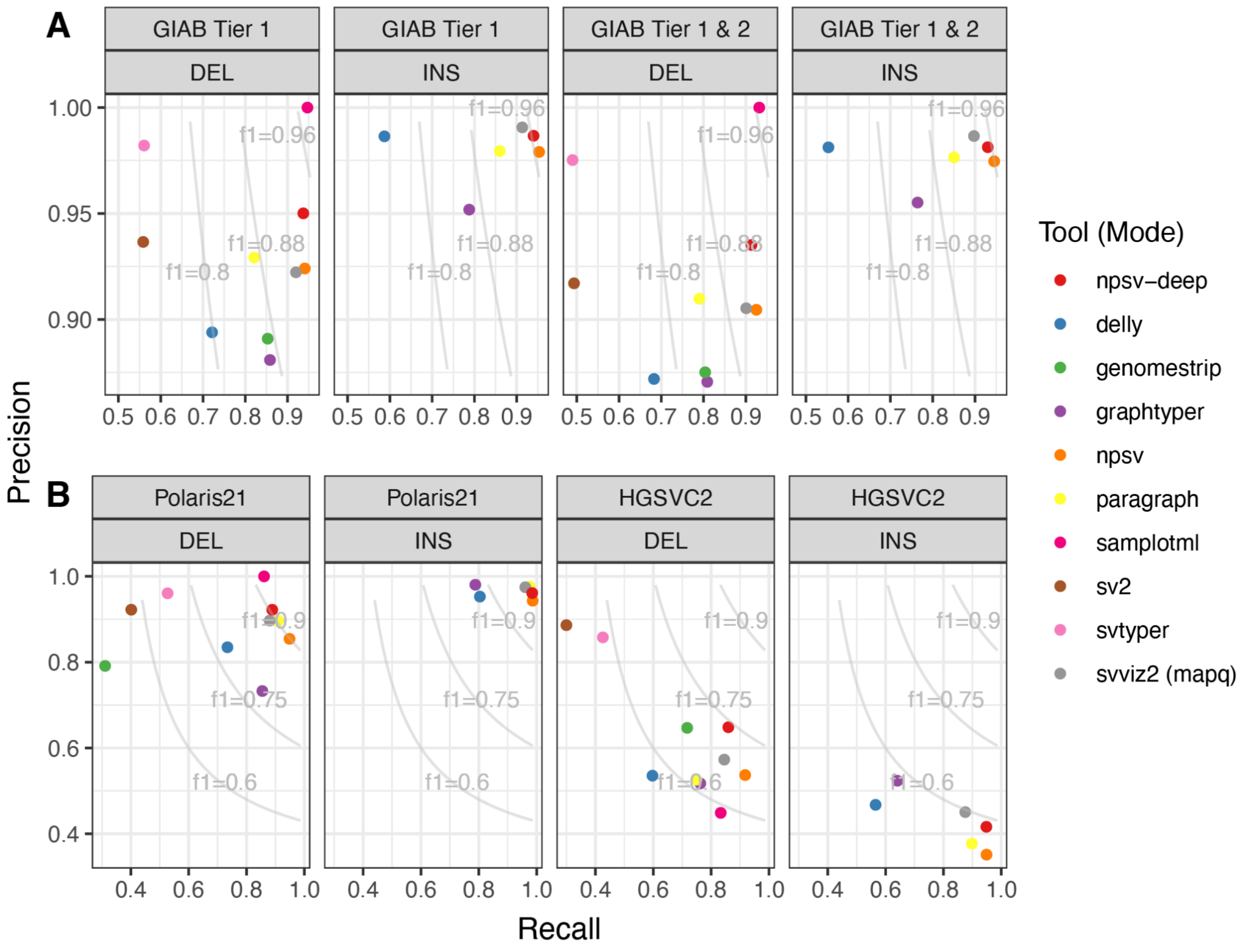

Supplemental Figure 2: Event-level metrics (presence vs. absence of SV) for HG002 and NA12878 SVs. An expanded version of Figure 3A.

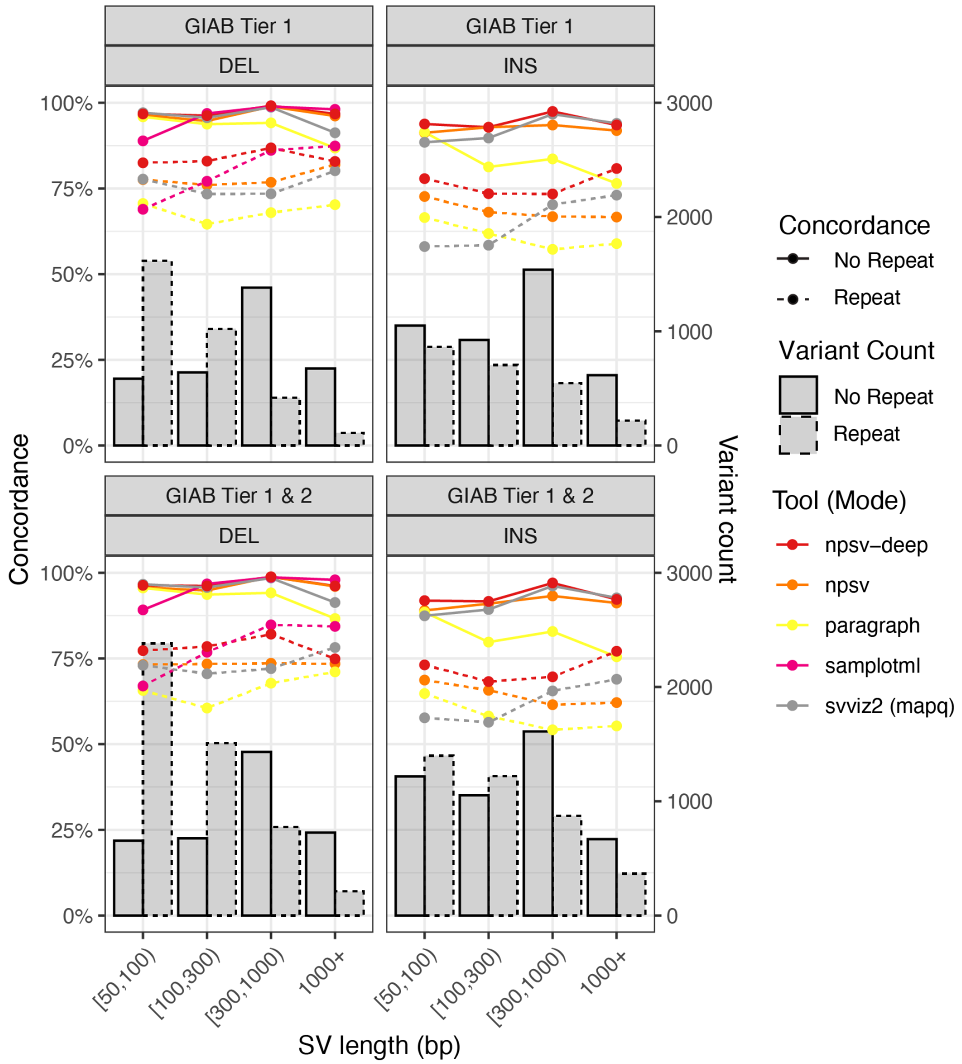

Supplemental Figure 3: Genotype concordance for SVs stratified by size and whether the SV overlaps a tandem repeat (TR) > 100 bp. The background bars show the counts of SVs of different sizes and repetitive contexts in the GIAB call set. An expanded version of Figure 3D.

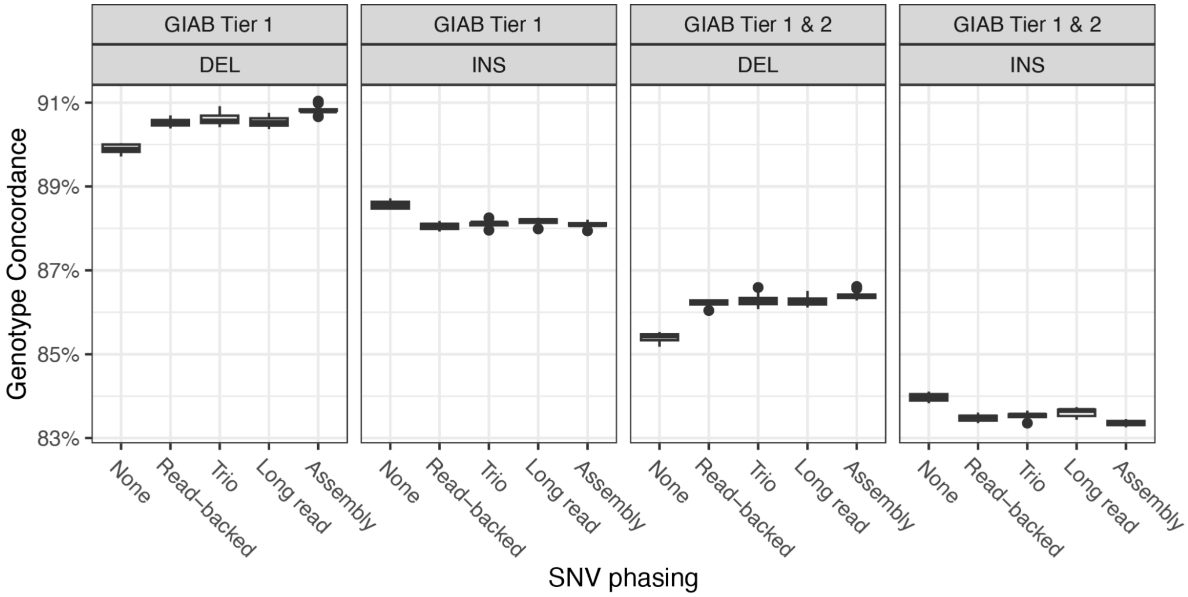

Supplemental Figure 4: Genotype concordance across 10 independent NPSV-deep genotyping runs for GIAB v0.6 SVs using different sources of phased SNVs. “None” uses the same DNN models, but does not provide SNVs during SV genotyping. “Read-backed” phasing uses WhatHap to phase HG002 SNVs using SRS data alone; unless otherwise noted, these are the results reported in the rest of the evaluation. “Trio” further incorporates SNVs called in the entire trio, “Long read” uses PacBio long reads for phasing and “Assembly” uses phased SNVs reported by HGSVC2.

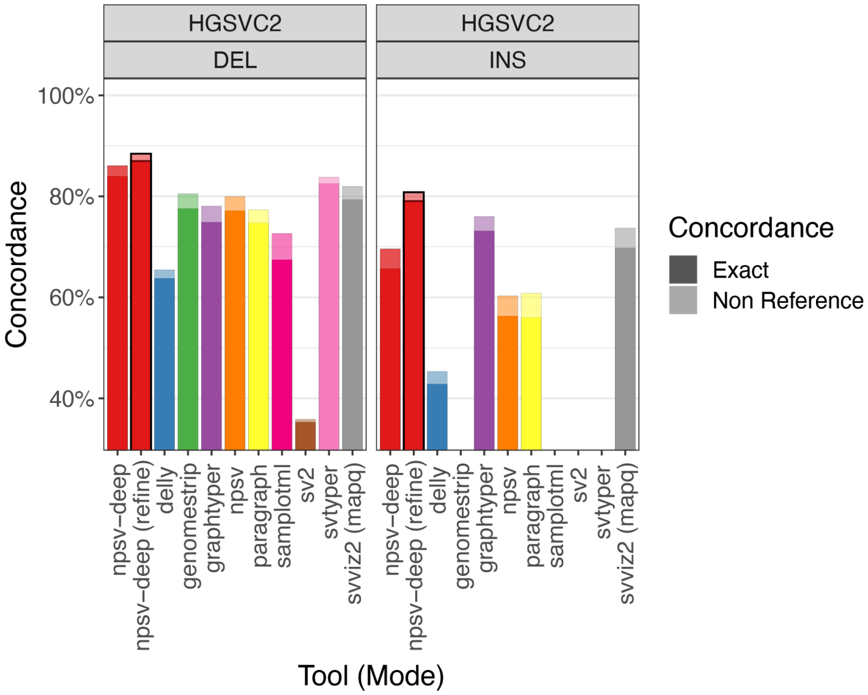

Supplemental Figure 5: Genotyping accuracy incorporating SV refining for HGSVC2 SVs in NA12878 using the Platinum Genomes SRS data.The “npsv-deep (refine)” workflow treats similar or overlapping SVs as “proposals”, applying the same refining algorithm to select the most likely SV for each group (predicting all others to be homozygous reference). The best concordance for each metric is highlighted with a black outline.

Supplemental Table 2: Counts of genotypes for SV truth sets used in concordance evaluation

**Dataset**			**Hom. Ref.**	**Het.**	**Hom. Alt.**
GIAB	Tier 1	DEL	2245	2560	1643
		INS	1018	2633	2810
	Tier 1 and 2	DEL	2905	3433	2030
		INS	1136	3502	3774
Polaris 2.1		DEL	17067	3463	2215
		INS	6294	2495	3430
HGSVC2		DEL	29058	5315	3347
		INS	48406	7387	6286

Supplemental Table 3: Exact (non-reference) genotype concordance for GIAB v0.6 SVs including “LongReadHomRef” SVs where “long reads supported homozygous reference for all individuals”) in tier 1 regions and tier 1 regions and tier 2 SVs combined. Best performance is bolded.

Tool	GIAB Tier 1 SVs		GIAB Tier 1 and 2 SVs	
	**DEL**	**INS**	**DEL**	**INS**
npsv-deep	**0.906** (0.927)	**0.881** (0.939)	**0.862** (0.902)	**0.834** (0.924)
delly	0.691 (0.711)	0.548 (0.595)	0.627 (0.664)	0.494 (0.559)
genomestrip	0.775 (0.834)		0.724 (0.795)	
graphtyper	0.775 (0.831)	0.638 (0.783)	0.726 (0.795)	0.594 (0.760)
npsv	0.873 (0.911)	0.842 (**0.943**)	0.832 (0.887)	0.794 (**0.931**)
paragraph	0.808 (0.840)	0.761 (0.864)	0.764 (0.810)	0.719 (0.850)
samplotml	0.857 (**0.965**)		0.831 (**0.955**)	
sv2	0.643 (0.662)		0.576 (0.600)	
svtyper	0.666 (0.706)		0.620 (0.658)	
svviz2 (mapq)	0.862 (0.897)	0.817 (0.920)	0.822 (0.874)	0.772 (0.901)

Supplemental Table 4: Exact (non-reference) genotype concordance for Polaris 2.1 and HGSVC2 call sets in NA12878 using Platinum Genomes SRS data. Best performance is bolded.

Tool	Polaris 2.1		HGSVC2	
	DEL	INS	DEL	INS
npsv-deep	**0.940** (0.954)	0.946 (0.973)	**0.840** (**0.861**)	0.657 (0.696)
delly	0.852 (0.864)	0.746 (0.794)	0.638 (0.654)	0.428 (0.453)
genomestrip	0.435 (0.449)		0.776 (0.805)	
graphtyper	0.860 (0.886)	0.854 (0.888)	0.749 (0.780)	**0.732** (**0.760**)
npsv	0.930 (0.947)	0.928 (0.964)	0.771 (0.799)	0.563 (0.603)
paragraph	0.935 (0.951)	**0.953** (**0.976**)	0.747 (0.773)	0.560 (0.608)
samplotml	0.921 (**0.965**)		0.674 (0.727)	
sv2	0.563 (0.570)		0.353 (0.358)	
svtyper	0.864 (0.874)		0.825 (0.838)	
svviz2 (mapq)	0.915 (0.945)	0.915 (0.969)	0.794 (0.820)	0.698 (0.737)

Supplemental Table 5: Exact (non-reference) genotype concordance for just private SVs excluded from the training set and all SVs in the HGSVC2 call set for N12878 using Platinum Genomes SRS data. The “All” columns are copied from Supplemental Table 4. Best performance is bolded.

**Tool**	**DEL**		**INS**	
	**Private (n=439)**	**All (n=37720)**	**Private (n=599)**	**All (n=62079)**
npsv-deep	**0.683 (0.747)**	**0.840** (**0.861**)	**0.599 (0.716)**	0.657 (0.696)
delly	0.556 (0.599)	0.638 (0.654)	0.345 (0.387)	0.428 (0.453)
genomestrip	0.667 (0.706)	0.776 (0.805)		
graphtyper	0.636 (0.706)	0.749 (0.780)	0.538 (0.601)	**0.732** (**0.760**)
npsv	0.677 (0.743)	0.771 (0.799)	0.559 (0.666)	0.563 (0.603)
paragraph	0.656 (0.702)	0.747 (0.773)	0.484 (0.624)	0.560 (0.608)
samplotml	0.654 (0.708)	0.674 (0.727)		
sv2	0.317 (0.330)	0.353 (0.358)		
svtyper	0.620 (0.631)	0.825 (0.838)		
svviz2 (mapq)	0.679 (0.738)	0.794 (0.820)	**0.599** (0.692)	0.698 (0.737)

Supplemental Table 6: Execution time and memory usage for Genome-in-a-Bottle Tier 1 and Tier 2 SVs on a 36-core compute node (dual 18-core Intel Xeon 6140 2.3 GHz CPUs). Execution time is determined with the time utility, and memory usage is the maximum resident set size reported by the SLURM cluster job queueing system. We ran tools that support parallel execution for a single sample on all 36 cores (using GNU Parallel[29] and the start/end range parameters to parallelize svviz2).

Tool	Phase	Time (HH:MM:SS)	Memory (b)	Cores
**Shared pre-processing (25.5× mean coverage BAM file)**				
NPSV-deep	preprocessing	2:24.01	1063928K	36
npsv	preprocessing	2:24:13	2018240K	1
**DEL SVs (*n*=8435)**				
NPSV-deep	genotyping	1:12:46	32081032K	36
delly	genotyping	25:01.97	3511856K	1
genomestrip	preprocessing	57:45.44	16644452K	36
	genotyping	21:41.18	16644452K	36
graphtyper	genotyping	14:49:21	5335532K	1
npsv	genotyping	14:47:41	21336752K	36
paragraph	genotyping	6:42.48	4246132K	36
samplotml	genotyping	17:06.68	4336184K	36
sv2	genotyping	29:50.82	681244K	1
svtyper	genotyping	2:10.48	1284840K	1
svviz2	genotyping	1:13:34	196980284K	36
	post-processing	0:48.86		
**INS SVs (*n*=8431)**				
NPSV-deep	genotyping	1:13:08	35224664K	36
delly	genotyping	24:39.69	3452024K	1
graphtyper	genotyping	14:51:18	5336992K	1
paragraph	genotyping	9:16.29	4178692K	36
npsv	genotyping	29:11.61	21432424K	36
svviz2	genotyping	44:30.39	195849584K	36
	post-processing	0:50.93		

References

1. Li, H. Aligning sequence reads, clone sequences and assembly contigs with BWA-MEM. Preprint at https://doi.org/10.48550/arXiv.1303.3997 (2013).
2. Wala, J. & Beroukhim, R. SeqLib: a C ++ API for rapid BAM manipulation, sequence alignment and sequence assembly. *Bioinformatics* **33**, 751–753 (2016).
3. Spies, N., Zook, J. M., Salit, M. & Sidow, A. svviz: a read viewer for validating structural variants. *Bioinformatics* **31**, 3994–6 (2015).
4. Martin, M. *et al.* WhatsHap: fast and accurate read-based phasing. Preprint at https://doi.org/10.1101/085050 (2016).
5. Poplin, R. *et al.* A universal SNP and small-indel variant caller using deep neural networks. *Nat. Biotechnol.* **36**, 983–987 (2018).
6. Wenger, A. M. *et al.* Accurate circular consensus long-read sequencing improves variant detection and assembly of a human genome. *Nat. Biotechnol.* **37**, 1155–1162 (2019).
7. Linderman, M. D. *et al.* NPSV: A simulation-driven approach to genotyping structural variants in whole-genome sequencing data. *GigaScience* **10**, giab046 (2021).
8. Huang, W., Li, L., Myers, J. R. & Marth, G. T. ART: a next-generation sequencing read simulator. *Bioinforma. Oxf. Engl.* **28**, 593–4 (2012).
9. Faust, G. G. & Hall, I. M. SAMBLASTER: fast duplicate marking and structural variant read extraction. *Bioinforma. Oxf. Engl.* **30**, 2503–5 (2014).
10. Chapman, B. *et al.* bcbio/bcbio-nextgen: v1.2.8. https://zenodo.org/record/4686097 Zenodo https://doi.org/10.5281/zenodo.4686097 (2021).
11. Quinlan, A. R. & Hall, I. M. BEDTools: a flexible suite of utilities for comparing genomic features. *Bioinforma. Oxf. Engl.* **26**, 841–2 (2010).
12. Li, H. *et al.* The Sequence Alignment/Map format and SAMtools. *Bioinforma. Oxf. Engl.* **25**, 2078–9 (2009).

13. Pedersen, B. S., Collins, R. L., Talkowski, M. E. & Quinlan, A. R. Indexcov: fast coverage quality control for whole-genome sequencing. *GigaScience* **6**, 1–6 (2017).

14. Koch, G., Zemel, R. & Salakhutdinov, R. Siamese neural networks for one-shot image recognition. in *Proceedings of the 32nd International Conference on Machine Learning* (Lille, France, 2015).

15. Martín Abadi *et al.* TensorFlow: Large-Scale Machine Learning on Heterogeneous Systems. https://www.tensorflow.org/ (2015).

16. Szegedy, C., Vanhoucke, V., Ioffe, S., Shlens, J. & Wojna, Z. Rethinking the Inception Architecture for Computer Vision. in 2818–2826 (IEEE, 2016). doi:10.1109/CVPR.2016.308.

17. Ebert, P. *et al.* Haplotype-resolved diverse human genomes and integrated analysis of structural variation. *Science* **372**, (2021).

18. Byrska-Bishop, M. *et al.* High-coverage whole-genome sequencing of the expanded 1000 Genomes Project cohort including 602 trios. *Cell* **185**, 3426-3440.e19 (2022).

19. Li, H. *et al.* A synthetic-diploid benchmark for accurate variant-calling evaluation. *Nat. Methods* **15**, 595–597 (2018).

20. Layer, R. M., Chiang, C., Quinlan, A. R. & Hall, I. M. LUMPY: a probabilistic framework for structural variant discovery. *Genome Biol.* **15**, R84–R84 (2014).

21. Chen, X. *et al.* Manta: rapid detection of structural variants and indels for germline and cancer sequencing applications. *Bioinformatics* **32**, 1220–1222 (2016).

22. Hansen, N. F. SVanalyzer. https://github.com/nhansen/SVanalyzer (2019).

23. Zook, J. M. *et al.* A robust benchmark for detection of germline large deletions and insertions. *Nat. Biotechnol.* **38**, 1347–1355 (2020).

24. Illumina. Polaris. https://github.com/Illumina/Polaris.

25. English, A. C., Menon, V. K., Gibbs, R. A., Metcalf, G. A. & Sedlazeck, F. J. Truvari: refined structural variant comparison preserves allelic diversity. *Genome Biol.* **23**, 271 (2022).

26. Danecek, P. *et al.* Twelve years of SAMtools and BCFtools. *GigaScience* **10**, (2021).

27. Pacific Biosciences. pbsv. https://github.com/PacificBiosciences/pbsv.

28. Robinson, J. T. *et al.* Integrative genomics viewer. *Nat. Biotechnol.* **29**, 24–26 (2011).

29. Tange, O. GNU Parallel 20220522 ('NATO’). https://zenodo.org/record/6570228 Zenodo https://doi.org/10.5281/zenodo.6570228 (2022).
